# Longitudinal association of gonorrhea and bacterial vaginosis with repeat chlamydia diagnoses among U.S. Army women: a retrospective cohort analysis

**DOI:** 10.1186/s40779-018-0184-3

**Published:** 2018-10-30

**Authors:** Christian T. Bautista, Eyako K. Wurapa, Warren B. Sateren, Bruce P. Hollingsworth, Jose L. Sanchez

**Affiliations:** 1 Division of Health Research, Lancaster University, Lancaster, UK; 2 Walter Reed Army Institute of Research, Silver Spring, MD USA; 3 Senior Research Consultant, Washington, DC USA; 4 Armed Forces Health Surveillance Branch, Public Health Division, Defense Health Agency, Silver Spring, MD USA

**Keywords:** Gonorrhea, Bacterial vaginosis, Chlamydia, Sexually transmitted infection, Military

## Abstract

**Background:**

Historically, sexually transmitted infections have affected the health of the U.S. military. To determine whether gonorrhea, bacterial vaginosis, genital herpes, and trichomoniasis are predictors of repeat chlamydia diagnoses among U.S. Army women, medical data reported into the Defense Medical Surveillance System during the 2006–2012 period were analyzed.

**Methods:**

For all inpatient and outpatient medical records, the first and second International Classification of Diseases, version 9 (ICD-9) diagnostic positions were reviewed for each chlamydia case to determine the occurrence of repeat diagnoses. The Andersen-Gill regression model, an extension of the Cox model for multiple failure-time data, was used to study associations between predictors and repeat chlamydia diagnoses.

**Results:**

Among 28,201 women with a first chlamydia diagnosis, 5145 (18.2%), 1163 (4.1%), 267 (0.9%), and 88 (0.3%) had one, two, three, and four or more repeat diagnoses, respectively. Overall, the incidence of repeat chlamydia was 8.31 cases per 100 person-years, with a median follow-up time of 3.39 years. Gonorrhea (hazard ratio (*HR*) = 1.58, 95% CI: 1.44–1.73) and bacterial vaginosis (*HR* = 1.40, 95% CI: 1.09–1.79) were significant predictors for repeat chlamydia. These estimated hazard ratios were attenuated, but remained significant, after controlling for age, race/ethnicity, marital status, and military rank. No significant association was found for genital herpes (*HR* = 1.13, 95% CI: 0.55–2.29) and trichomoniasis (*HR* = 1.43, 95% CI: 0.43–4.68).

**Conclusions:**

This large cohort study suggests that gonorrhea and bacterial vaginosis were associated with repeat chlamydia diagnoses among U.S. Army women. These findings can be used in formulating new interventions to prevent repeat chlamydia diagnoses.

## Background

In the U.S. civilian population, chlamydia is a commonly diagnosed sexually transmitted infection (STI) [1]. In 2014, approximately 1.4 million cases of chlamydia were reported, of which, most occurred among young people and women. Chlamydia also represents a significant health problem for the U.S. military. According to the Armed Forces Health Surveillance Branch, during the period 2000–2012, 198,274 chlamydia diagnoses were reported among service members (SMs) [2]. Over 44% of these diagnoses occurred in women SMs and 39% in African-American personnel.

In women, chlamydia can be associated with reproductive complications such as pelvic inflammatory disease and ectopic pregnancy. However, the risk of such complications is much higher in women with repeat chlamydia infection [3]. According to the literature, no single behavioral risk factor has been consistently reported to predispose to repeat infection [3, 4]. In part, this is because the mechanisms underlying repeat infections are complex in the field of STIs. In addition, in most studies of repeat chlamydia, the event of interest has been time to the first repeat infection, ignoring the subsequent infections. Therefore, those studies are suboptimal to address the true burden of repeat chlamydia.

The literature also indicates a high rate of repeat chlamydia among U.S. military women. During 1994–1998, the rate of repeat chlamydia among U.S. Army women was 65.79 per 1000 person-years (py) [5]. In another study conducted at Fort Bragg (the largest U.S. Army base worldwide in terms of population) during 2005–2010, the rate of repeat chlamydia in women was 161.9 per 1000 py [6]. Recently, it has been estimated that, during 2010–2014, the rate of repeat chlamydia was 116.3 per 1000 py among U.S. military women [7]. Although these studies offer insights into the epidemiology of repeat chlamydia in the U.S. military, only risk factors, such as younger age, black race, and single marital status have been studied; other risk factors, such as gonorrhea, have not been studied [8].

Given the paucity of studies examining whether other STIs and common gynecological disorders represent potential risk predictors for repeat chlamydia in the U.S. military, the aim of this retrospective cohort study was to characterize whether gonorrhea, genital herpes, trichomoniasis or bacterial vaginosis (BV) were associated with repeat chlamydia among female SMs. The findings can be applied to guide further targeted STI research and to implement new prevention efforts to reduce the burden of chlamydia in U.S. Army women.

## Methods

### Data source

This study used personnel and medical data registered in the Defense Medical Surveillance System (DMSS). The DMSS is the central health surveillance system for recording and monitoring the health of the U.S. Armed Forces. This surveillance system (over 2 billion medical records by 2015) collects data regarding all medical encounters (in-patient and out-patient), including immunizations, reportable events, and human immunodeficiency virus (HIV) test results. It also collects personnel demographic and deployment data for service members from the day they enter the service until the day they leave. At clinic and hospital sites, the medical staff input data from the patient medical charts into the Armed Forces Health Technology Application, which is then transferred to the DMSS. Using the International Classification of Diseases, version 9 (ICD-9), for each medical event, a maximum of eight codes can be registered. The first two codes register the primary reasons for the visit. Details about the DMSS have been published elsewhere [9].

According to a survey of the U.S. Army laboratories (31 in the U.S. and 7 in Europe and Asia), from the 255,509 *C. trachomatis* specimens collected in 2007, 79% were tested using nucleic acid amplification tests (NAATs) [10]. This survey also revealed that NAATs followed by culture were the most common methods utilized to detect *N. gonorrhoeae*. Similar findings were recently published by Leamer et al [11]. In the U.S. Army, diagnosis of BV and trichomoniasis is usually based on the four Amsel criteria and wet mount examination, respectively.

### Study design and population

The study population consisted of women personnel who were on active duty in the U.S. Army for any period between January 1, 2006, and December 31, 2012. All women with a chlamydia diagnosis during the study period, and without a chlamydia diagnosis prior to January 1, 2006, were selected. The criteria for a diagnosis of chlamydia was based on the ICD-9 diagnostic codes. For chlamydia diagnosis, the ICD-9 codes were 099.41 or 099.5. For all inpatient and outpatient medical records, the first and second diagnostic position were reviewed for each chlamydia case to determine the occurrence of repeat diagnoses until the end of the study period or termination of military service. Given than NAATs can detect residual bacterial deoxyribonucleic acid for up to 3 weeks after therapy [12], a repeat chlamydia diagnosis was defined as one which occurred 30 days after the previous diagnosis. The decision to use 30 days for determining repeat chlamydia also followed the recent Centers for Disease Control and Prevention (CDC) guidelines [13].

### Study variables

Demographic variables included age, race/ethnicity (white, African-American, other), educational level (no high school, high school or higher), marital status (single, married, other), and military rank (officer, enlisted). To determine whether other STIs or common gynecological disorders increased the hazard of repeat chlamydia diagnoses, the DMSS database was searched using ICD-9 codes for gonorrhea (098.0×, 098.1×, 098.4×, or 098.8×), BV (616.10), genital herpes simplex virus (054.1), and trichomoniasis (131.9). Women with these ICD-9 codes before or at the time of their first chlamydia diagnosis were excluded from analysis. The rationale for this exclusion criteria was to exclude women SMs with previous infections from the study population, to prevent confusion of the results.

### Statistical analyses

The chi-square test was used to compare the percentage of gonorrhea and BV among women with or without repeat chlamydia diagnoses. The rate of repeat chlamydia diagnoses was estimated by the number of repeat diagnoses divided by the total follow-up period per 100 py. To determine the association between repeat chlamydia diagnoses and other STIs and BV, the counting process (multiple observations are created for each individual) of the Andersen-Gill regression model was applied [14]. This model, which is an extension of the Cox regression method for analyzing data with repeated events, assumes that the correlation among events is induced by measured covariates, which is the aim of this study [15]. In the regression models, gonorrhea, BV, genital herpes, and trichomoniasis were treated as time-varying variables, whereas demographic and military characteristics were treated as fixed variables. The time scale was days and the time-varying variables were updated at the respective entry times. Associations were expressed in terms of hazard ratios (*HR*) with 95% confidence intervals (95% CI). All analyses were performed using Stata version 13.1 (Stata Corporation, College Station, TX).

## Results

During the study period, 37,419 women SMs with a first chlamydia diagnosis were registered into the DMSS. Of these, 9218 (25%) were excluded due to the exclusion criteria. Among the eligible population (*n* = 28,201, 75%) as shown in Table 1, 89% of women SMs were enlisted members, 84% were 17–24 years old (*mean* = 21.6 years), 73% were single, and 43% were white. Additionally, most of the population had a high school or higher education (93%).

**Table 1:** Demographic characteristics and repeat chlamydia diagnoses among U.S. army women

Item	Women with initial diagnosis of chlamydia (*n*)	Women with repeat diagnoses of chlamydia (*n* (%))			Incidence rate of repeat chlamydia diagnoses^c^	
		One	Two	Three or more	Cases per 100 py (*n*)	95% CI
All women	28,201	5145 (18.2)	1163 (4.1)	355 (1.3)	8.31	8.14, 8.49
Age group (year)						
17–19	8540	1959 (22.9)	532 (6.2)	179 (2.1)	11.56	11.19, 11.95
20–24	15,319	2687 (17.5)	558 (3.6)	159 (1.0)	7.73	7.50, 7.96
>=25	4342	499 (11.5)	73 (1.7)	17 (0.4)	4.15	3.86, 4.47
Race/Ethnicity						
White	12,046	1983 (16.5)	364 (3.0)	95 (0.8)	7.25	6.99, 7.51
African-American	8605	1783 (20.7)	480 (5.6)	166 (1.9)	9.80	9.47, 10.13
Other^a^	7112	1304 (18.3)	304 (4.3)	84 (1.2)	8.15	7.82, 8.50
Marital status						
Single	20,487	3965 (19.3)	924 (4.5)	282 (1.4)	8.87	8.66, 9.09
Married	6349	981 (15.4)	210 (3.3)	65 (1.0)	7.08	6.74, 7.43
Other^b^	1354	197 (14.5)	29 (2.1)	8 (0.6)	5.41	4.81, 6.08
Military rank						
Enlisted	25,125	4797 (19.1)	1120 (4.5)	346 (1.4)	9.02	8.83, 9.22
Officers	3076	348 (11.3)	43 (1.4)	9 (0.3)	3.45	3.15, 3.78

*CI* Confidence interval, Denominators may vary due to missing data. ^a^Other: Hispanic, American Indian/Alaskan Native, Asian/Pacific Islander, and others. ^b^Other: Divorced, widowed, and others. ^c^ Rate reported as per 100 person-years

The median follow-up was 3.39 years (interquartile range (*IQR*) = 2.07–5.26 years). During the follow-up period, 6663 (23.6%) women had at least one repeat chlamydia diagnosis. Of these, 5145 (18.2%), 1163 (4.1%), and 355 (1.3%) had one, two, and three or more repeat diagnoses, respectively. Overall, the rate of repeat chlamydia diagnoses was 8.31 cases (95% CI: 8.14–8.49) per 100 py. Higher repeat rates occurred among those women who were younger (17–19 years old), of African-Americans race/ethnicity, in the enlisted ranks, and single (Table 1).

Among 28,201 women personnel, the percentage of gonorrhea, BV, genital herpes, and trichomoniasis was 2.6% (*n* = 735), 0.5% (*n* = 150), 0.1% (*n* = 19), and 0.04% (*n* = 12), respectively. For women with repeat chlamydia diagnoses, gonorrhea and BV percentages were significantly higher compared to women without repeat chlamydia diagnoses (Table 2). Significant differences were also noted between the women with and without repeat chlamydia diagnoses in terms of age group, race/ethnicity, marital status, and military rank subcategories. Regarding genital herpes and trichomoniasis, their percentages were 0.15% (*n* = 10) and 0.12% (*n* = 8), respectively, for women with repeat chlamydia diagnoses, and 0.04% (*n* = 9) and 0.02% (*n* = 4), respectively, for women without repeat chlamydia diagnoses. Due to the small number of women with a diagnosis of genital herpes or trichomoniasis, data by subcategories are not shown.

**Table 2:** Percentage of gonorrhea and bacterial vaginosis among women with or without repeat chlamydia diagnoses among U.S. Army women (%)

Feature	Women with repeat chlamydia diagnoses			Women without repeat chlamydia diagnoses		
	Gonorrhea^c^	BV^c^	*P* value	Gonorrhea^c^	BV^c^	*P* value
All women	6.9 (459/6663)	1.3 (88/6663)	< 0.001	1.3 (276/21,538)	0.3 (62/21,538)	< 0.001
Age group (year)						
17–19	8.3 (221/2670)	1.3 (34,2670)	< 0.001	1.7 (98/5870)	0.3 (20/5870)	< 0.001
20–24	6.3 (213/3404)	1.3 (43/3404)	< 0.001	0.5 (148/11,915)	0.3 (35/11,915)	< 0.001
>= 25	4.2 (25/589)	1.9 (11/589)	< 0.001	0.8 (30/3753)	0.2 (7/3753)	< 0.001
Race/Ethnicity						
White	3.9 (96/2442)	1.0 (24/2442)	< 0.001	0.7 (63/9604)	0.2 (24/9604)	< 0.001
African-American	11.2 (272/2429)	1.9 (47/2429)	< 0.001	3.0 (166/6176)	0.3 (20/6176)	< 0.001
Other^a^	5.1 (86/1692)	1.0 (17/1692)	< 0.001	0.8 (44/5420)	0.3 (16/5420)	< 0.001
Marital Status						
Single	7.4 (384/5171)	1.2 (64/5171)	< 0.001	1.3 (203/15,316)	0.3 (46/15,316)	< 0.001
Married	5.0 (63/1256)	1.7 (21/1256)	< 0.001	1.2 (61/5093)	0.3 (14/5093)	< 0.001
Other^b^	5.1 (12/234)	1.3 (3/234)	< 0.001	1.2 (11/1120)	0.2 (2/1120)	0.011
Military rank						
Enlisted	7.2 (449/6263)	1.3 (83/6263)	< 0.001	1.4 (266/18,862)	0.3 (59/18,862)	< 0.001
Officers	2.5 (10/400)	1.2 (5/400)	< 0.001	0.4 (10/2676)	0.1 (3/2676)	< 0.001

*BV* Bacterial vaginosis. ^a^Other: Hispanic, American Indian/Alaskan Native, Asian/Pacific Islander, and others, ^b^Other: Divorced, widowed, and others

^c^At least one episode

According to the Andersen-Gill regression analysis, gonorrhea (*HR* = 1.58, 95% CI: 1.44–1.73) and BV (*HR* = 1.40, 95% CI: 1.09–1.79) were significantly associated with repeat chlamydia diagnoses (Table 3). After controlling for age (in years), the adjusted *HR*s for gonorrhea (*HR* = 1.47) and BV (*HR* = 1.32) were attenuated but remained significant (Model 1). Similar adjusted *HR* were obtained after controlling for age, race/ethnicity, marital status, and military rank (Model 2). With respect to genital herpes and trichomoniasis, although these STIs were positively associated with repeat chlamydia diagnoses, their HRs were not significant in univariate and multiple analyses.

**Table 3:** Unadjusted and adjusted hazard ratios for repeat chlamydia diagnoses among U.S. Army women

Feature	Unadjusted		Adjusted Model 1^a^		Adjusted Model 2^b^	
	Crude *HR*	95% CI	Adjusted *HR*	95% CI	Adjusted *HR*	95% CI
Gonorrhea	1.58	1.44, 1.73^*^	1.47	1.34, 1.62^*^	1.46	1.33, 1.60^*^
Bacterial vaginosis	1.40	1.09, 1.79^**^	1.32	1.03, 1.69^**^	1.31	1.04, 1.68^**^
Genital herpes	1.13	0.55, 2.29	1.04	0.53, 2.04	1.03	0.52, 2.06
Trichomoniasis	1.43	0.43, 4.68	1.16	0.35, 3.88	1.13	0.34, 3.75
At least one of above	1.69	1.53, 1.88^*^	1.54	1.39, 1.71^*^	1.53	1.38, 1.69^*^

^a^Adjusted for age (years); ^b^Adjusted for age (years), race/ethnicity, marital status, and military rank. ^*^P < 0.001; ^**^P < 0.05

An analysis stratified by the number of repeat gonorrhea diagnoses indicated that, among the 735 women with gonorrhea, 561 had one diagnosis, 142 two diagnoses, 23 three diagnoses, and 9 four or more diagnoses. For each additional diagnosis of gonorrhea, the hazard of repeat chlamydia diagnosis increased significantly by 58% (95% CI: 44–73%). After controlling for age, race/ethnicity, marital status, and military rank, the adjusted hazard for repeat chlamydia was 46% (95% CI: 33–60%).

## Discussion

The findings from this large retrospective cohort analysis revealed a high incidence of repeat chlamydia, and that gonorrhea and BV were significant predictors associated with repeat chlamydia among U.S. Army women.

Our estimated repeat chlamydia rate (8 diagnoses per 100 py) was higher than that reported by Barnett and Brundage [5] (6 diagnoses per 100 py) and lower compared to Hakre et al [6] (16 diagnoses per 100 py). Some methodological differences in the design and analysis may explain these differences. First, the above studies determined the rate of recurrence to the first diagnosis, ignoring all subsequent diagnoses. Second, Barnett and Brundage [5] used only one ICD-9 code (099.41) to identify chlamydia diagnoses. Finally, we excluded approximately 25% of women with a history of STI, which may have affected our estimates given that an STI history is a predictor for repeat chlamydia [3, 16]. Nonetheless, these studies indicate a high rate of repeat chlamydia among U.S. Army women.

It was also observed that the highest repeat chlamydia rates occurred among young women (17–19 years), African-Americans, and single women. These observations are consistent with the literature [3]. In a study of 38,866 women, Hillis et al [8] reported that younger age (< 20 years) was a strong predictor for repeat chlamydia. A secondary data analysis from a large trial also determined that black women were at high risk of repeat chlamydia [17]. Additionally, a retrospective cohort study reported that single women had 1.60 times the risk of repeat chlamydia compared to married women [16]. In addition, the finding that enlisted personnel, compared to officers, had a higher rate of repeat chlamydia has been previously reported in the military literature [5, 7].

The analysis also revealed that the percentage of gonorrhea among women with repeat chlamydia was higher compared to women without repeat chlamydia. This finding suggests that, despite the decrease in the annual gonorrhea rates among women SMs during the study period [2], this bacterial STI has an impact on the burden of chlamydia. Similarly, BV was more prevalent among women with repeat chlamydia than women without repeat chlamydia. Interestingly, we found that, compared to single women, married women had a higher percentage of BV. While this observation contradicts the literature on BV, the higher incidence may be associated with a higher frequency of vaginal intercourse among married women. Based on the studies of Leppäluoto et al [18] and Vallor et al [19], who reported that higher frequencies of vaginal intercourse cause loss of hydrogen peroxide and replacement of the dominant lactobacilli microbiota by *G. vaginalis* before and after intercourse, Verstraelen et al [20] have argued that the associated risk factors for BV (e.g., multiple sexual partners) are proxy variables to the true risk exposure, which is the frequency of vaginal intercourse.

In our study, gonorrhea was the strongest diagnostic predictor for repeat chlamydia. Two large studies have documented that women with gonorrhea are at increased risk of recurrent chlamydia [8, 21]. In both studies, gonorrhea was evaluated at the time of initial chlamydia infection, and therefore, their results represent the risk of coinfection. Moreover, their analyses ignored the correlation between repeat events. If this correlation is ignored, the confidence intervals for the regression coefficients can be biased [22]. By contrast, in our analyses, gonorrhea was treated as a time-varying covariate, which is an appropriate approach to study repeated events. To help better understand the association between gonorrhea and repeat chlamydia, coinfected women or women with a history of gonorrhea before the initial chlamydia diagnosis were excluded from analysis. Our results strengthen the evidence that gonorrhea and repeat chlamydia are associated.

Additionally, we found that a diagnosis of BV was a predictor for repeat chlamydia among U.S. Army women. Although several studies have indicated an association between BV and chlamydia, including a recent large military study published by the authors [23], no study in the STI literature has reported that BV is associated with an increased risk of repeat chlamydia. This finding needs to be confirmed in further studies. There is ongoing debate as to whether BV is an STI or is simply a sexually enhanced disease [20]. The mechanisms for the relationship between chlamydia and BV are unclear given the lack of a definitive etiologic agent for this condition. With respect to genital herpes and trichomoniasis, it was not possible to obtain reliable HR estimates due to the small number of women with these STIs. Whether or not the burden of genital herpes and trichomoniasis is, in fact, low among female chlamydia “repeaters” in the U.S. Army should be confirmed through further research.

In military STI research, there are studies suggesting that risky sexual behaviors such as inconsistent condom use, having many sexual partners, unwanted sexual contact, and engaging in sex under the influence of alcohol are risk factors associated with chlamydia among women SMs [24, 25]. However, there is a lack of information on what sexual risk behaviors are related to repeat chlamydia. Due to limitations of the DMSS data (e.g., no collection of data on sexual behaviors), it is not possible to determine whether repeat chlamydia diagnoses is due to reinfection either from an infected new sexual partner or from an untreated pre-existing sexual partner. Considering that U.S. Army women are predominantly young and are more likely to engage in high-risk sexual behaviors, it is plausible to consider that repeat chlamydia infections are influenced, to a significant extent, by reinfection from an untreated sexual partner, either civilian or military.

Identification of the risk predictors for initial and repeat chlamydia will help with clinical STI prevention and intervention efforts. Our findings contribute to the growing body of literature that is useful to clinicians while offering advice to female SMs with chlamydia; female SMs need to be retested 3 months after completion of treatment, regardless of whether their sexual partners were treated. This suggestion is consistent with the CDC’s recommendations. In addition, chlamydia “repeaters” should receive comprehensive sex education and risk reduction counselling (e.g., consistent and correct usage of condoms) during their health care visits to prevent further reinfection. Additionally, chlamydia “repeaters” should be educated that this common infection is associated with adverse reproductive health outcomes, such as preterm birth and gynecological complications, including pelvic inflammatory disease. Further, it is useful for the U.S. Army authorities to determine whether military physicians are following the CDC’s recommendations for chlamydia and other common STIs.

In the field of STIs, several studies have reported that the clinical practice of treating the sexual partners of patients diagnosed with chlamydia, also known as expedited partner therapy (EPT), has reduced the incidence of reinfections [26]. However, to date, the U.S. military has not implemented this evidence-based approach for the management of chlamydia, gonorrhea, or any STI [27]. In part, this is due to the need for establishing close coordination with local civilian health departments because service members may have civilian partners. In addition, other medical and legal issues relating to the implementation of a military EPT program require consideration.

This study had some limitations. First, as mentioned previously, due to the absence of sexual behavior data (e.g., number of sexual partners) or travel-related risks in the DMSS, our estimates are subjected to confounding bias, which is an inherent problem of utilizing data from electronic medical systems. Second, the length of follow-up in our study may have impacted the number of repeat chlamydia diagnoses. Third, because gonorrhea and chlamydia are mostly asymptomatic among women, it is possible that the burden of repeat chlamydia in our population is underestimated. Fourth, the DMSS data cannot be utilized to determine whether repeat chlamydia is due to treatment failure, and therefore, over-reporting of chlamydia diagnoses is possible; the estimated cure rate of azithromycin and doxycycline for *C. trachomatis* is 97% and 98%, respectively [28]. Fifth, although the U.S. military follows the CDC’s STI treatment guidelines, it was not possible to determine whether antibiotic treatment for chlamydial and gonococcal infections was consistent during the study period. Lastly, the associations may be biased due to misclassification of BV and trichomoniasis by employing the Amsel criteria method and wet mount inspection, respectively. Despite these limitations, we provide evidence for additional clinical research and generate new hypotheses for further military investigation.

## Conclusions

Based on the DMSS data and Andersen-Gill regression analysis, gonorrhea and BV were significant predictors for repeat chlamydia diagnoses among U.S. Army women during the 2006–2012 study period. Women personnel with repeat chlamydia represent a major reservoir for the ongoing transmission of infection, and reinfection increases the risk of further medical and reproductive complications. Our findings can guide military authorities in developing STI prevention programs to reduce the burden of “repeat” chlamydia infection in the U.S. military. As the U.S. Army has a population composed of members from differing cultural and socioeconomic backgrounds, culturally appropriate sex education programs are required and may be beneficial in terms of public health.

## Abbreviations

BV: Bacterial vaginosis
DMSS: Defense Medical Surveillance System
ICD-9: International Classification of Diseases, version 9
NAATs: Nucleic acid amplification tests
STI: Sexually transmitted infection
